# Complete Resolution of Pityriasis Rubra Pilaris With Targeted Treatment: A Case Report

**DOI:** 10.7759/cureus.82660

**Published:** 2025-04-20

**Authors:** Khadijah H Muzaffar, Abdullah Albadri

**Affiliations:** 1 General Practice, Batterjee Medical College, Jeddah, SAU; 2 Dermatology, King Fahad General Hospital, Jeddah, SAU

**Keywords:** dermatosis, pityriasis rubra pilaris, skin, treatment, ustekinumab

## Abstract

Pityriasis rubra pilaris (PRP) is a rare inflammatory dermatosis characterized by erythematous plaques with follicular hyperkeratotic papules, presenting challenges in diagnosis and management. Here, we present a case report of a 70-year-old male with a known medical history of diabetes mellitus and hypertension who presented with a two-year history of pruritic, purple papules, plaques, and scaly lesions distributed symmetrically on the hands, chest, and legs. Histological examination confirmed PRP, revealing classic features of PRP, including broad epidermal ridges with thick suprapapillary plates and perivascular lymphocytic infiltrate in the dermis. Treatment with systemic retinoids initially provided partial improvement; however, ustekinumab, a monoclonal antibody targeting interleukin-12 and -23, led to significant clinical improvement. This case highlights the therapeutic potential of ustekinumab in refractory PRP cases and the importance of histopathological confirmation in guiding treatment decisions.

## Introduction

Pityriasis rubra pilaris (PRP) is a chronic inflammatory papulosquamous skin disease, with an estimated incidence of 1 per 5,000 dermatologic patients in the United Kingdom, although the precise population-based prevalence remains unknown [1]. While PRP affects both sexes equally, it exhibits a bimodal age distribution, with peaks in the first and fifth decades of life [2]. The characteristic features of PRP include palmoplantar keratoderma (thickening of the skin on palms and soles)and follicular hyperkeratotic papules, which gradually coalesce into orange-red scaly plaques. These plaques typically exhibit well-demarcated areas of uninvolved skin, often described as *islands of sparing* or *nappes claires* [1]. Due to its chronic nature and noticeable PRP lesions, PRP can significantly impact patients' psychological well-being, social interactions, and overall quality of life [2].

Griffiths introduced a classification system in 1980, categorizing PRP into five subtypes. Subsequently, Miralles et al. expanded this classification in 1995 to encompass a sixth subtype, which is characterized by concurrent human immunodeficiency virus (HIV) infection and a poorer prognosis, as described in previous classification studies [1-3].

Management of PRP involves tailored approaches based on disease extension and severity. Topical therapy is recommended for localized forms, such as PRP type III, and is often used adjunctively with systemic therapy for severe cases to alleviate cutaneous symptoms. Medium to high potency corticosteroids, keratolytics, emollients, and Vitamin D derivatives are commonly employed for localized forms. Systemic treatment is reserved for moderate-to-severe disease, with retinoids being the mainstay therapy. While most cases respond favorably to retinoids, refractory cases pose a challenge, with limited standardized treatment protocols available due to the rarity of PRP. In recent years, biological agents have emerged as promising options for refractory cases, targeting cytokines implicated in PRP pathogenesis such as TNF-alpha and IL-17. Phototherapy, including ultraviolet B (UVB) and psoralen-ultraviolet A (PUVA) therapy, may also be effective, although responses vary among patients. Methotrexate and other immunosuppressants are considered alternatives for second-line treatment, while newer therapies like apremilast and extracorporeal photochemotherapy show promise in select cases. The management of PRP requires a tailored approach considering disease severity, treatment response, and potential adverse effects, with ongoing research needed to establish standardized treatment guidelines [4].

In this paper, we report a PRP case of a 71-year-old male with improved status due to ustekinumab treatment.

## Case presentation

**Figure 1:**
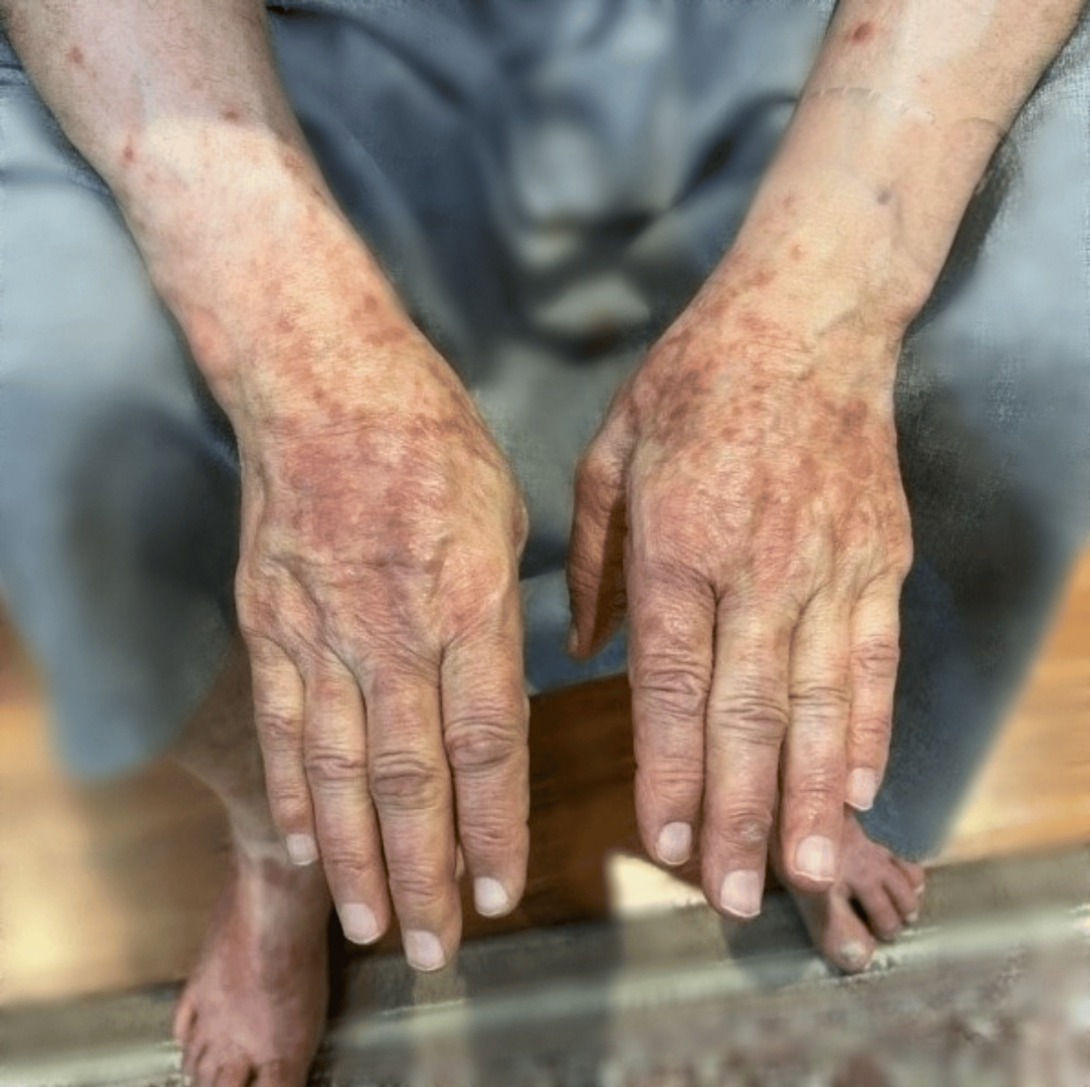
Multiple erythematous, scaly plaques and papules on the dorsal aspect of the hands and fingers. Some papules are extended to the right forearm.

The patient was a 70-year-old male with a known medical history of diabetes mellitus (DM) and hypertension (HTN). On February 12, 2023, the patient presented with complaints of pruritic, purple papules, plaques, and scaly lesions on the hands, chest, and both legs for two years (Figure 1).

The differential diagnosis (DDX) considered included mycosis fungoides (MF), psoriasis, lichen planus, and PRP. A skin biopsy was performed to further evaluate the patient's condition. Additionally, a purified protein derivative (PPD) test was conducted on the first visit, with a follow-up scheduled in two days to review the test reading. The PPD test result was negative, with 0 mm induration observed. The serological markers were negative except for Hepatitis B surface antibody (HBsAb) (Table 1). The initial biochemistry workup revealed some abnormalities, notably elevated creatinine and BUN levels, and eosinophilia (Table 1). The patient's initial laboratory findings are summarized in Table 1. Treatment with soft white paraffin (Vaseline) applied topically three times daily and calcipotriol 50 mcg + betamethasone ointment 0.5 mg ointment applied once daily (OD) was initiated to address the patient's skin dryness and inflammation symptoms pending further evaluation.

**Table 1:** Laboratory investigations. LDL, low-density lipoprotein; HDL, high-density lipoprotein

Parameters	Patient values	Reference range
Biochemistry		
Blood urea nitrogen (BUN)	38 mg/dL	6-20 mg/dL
Creatinine	1.74 mg/dL	0.8-1.3 mg/dL
LDL cholesterol	93.74 mg/dL	<100 mg/dL
Triglycerides	151.3 mg/dL	<150 mg/dL
Aspartate aminotransferase (AST)	20.7 U/L	0-37 U/L
HDL cholesterol	39.3 mg/dL	>40 mg/dL
Alanine aminotransferase (ALT)	28.5 U/L	0-63 U/L
Total cholesterol	163.3 mg/dL	<200 mg/dL
Hematology (CBC and differential)		
White blood cell (WBC) count	8.86 ×10⁹/L	4-10 ×10⁹/L
Red blood cell (RBC) count	4.17 ×10¹²/L	4.5-5.5 ×10¹²/L
Hemoglobin (HGB)	13 g/dL	13-17 g/dL
Hematocrit (HCT)	41.1%	40%-50%
Mean corpuscular volume (MCV)	98.7 fL	83-101 fL
Mean corpuscular hemoglobin (MCH)	31.2 pg	27-32 pg
Mean corpuscular hemoglobin concentration (MCHC)	31.6 g/dL	31.5-34.5 g/dL
Platelet count	256 ×10⁹/L	150-410 ×10⁹/L
Differential segmented neutrophils (%)	56.5%	40%-80%
Differential eosinophils (%)	18.1% (High)	1%-6%
Differential basophils (%)	0.937% (Low)	1%-2%
Differential monocytes (%)	8.45%	2%-10%
Differential lymphocytes (%)	16% (Low)	20%-40%
RDW-CV	13.2%	11.6%-14%
Absolute neutrophils (Seg)	4.89 ×10⁹/L	2-7 ×10⁹/L
Absolute eosinophils (Eos)	1.57 ×10⁹/L (High)	0.02-0.5 ×10⁹/L
Absolute basophils (Baso)	0.081 ×10⁹/L	0.02-0.1 ×10⁹/L
Absolute monocytes (Mono)	0.732 ×10⁹/L	0.2-1 ×10⁹/L
Absolute lymphocytes (Lymph)	1.38 ×10⁹/L	1-3 ×10⁹/L
Serology		
Hepatitis B surface antibody (HBsAb)	Positive	Negative
HIV antibody (HIV Ab)	Negative	Negative
Hepatitis C virus antibody (HCV Ab)	Negative	Negative
Hepatitis B surface antigen (HBsAg)	Negative	Negative

**Figure 2:**
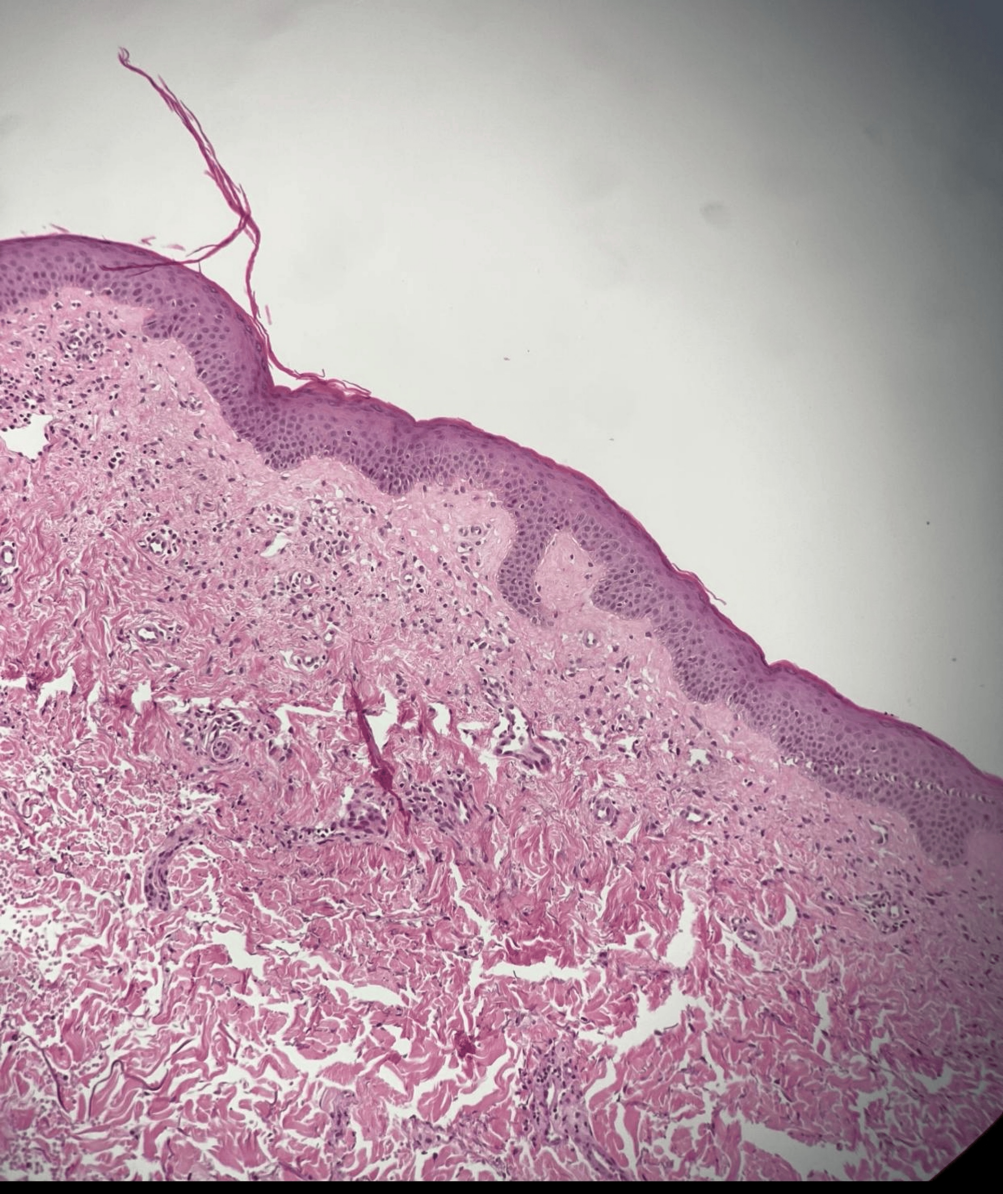
Histopathological features of PRP. Histopathological image showing flaky hyperkeratosis; mild epidermal acanthosis with broad rete ridges and thick suprapapillary plates (low magnification). PRP, pityriasis rubra pilaris

**Figure 3:**
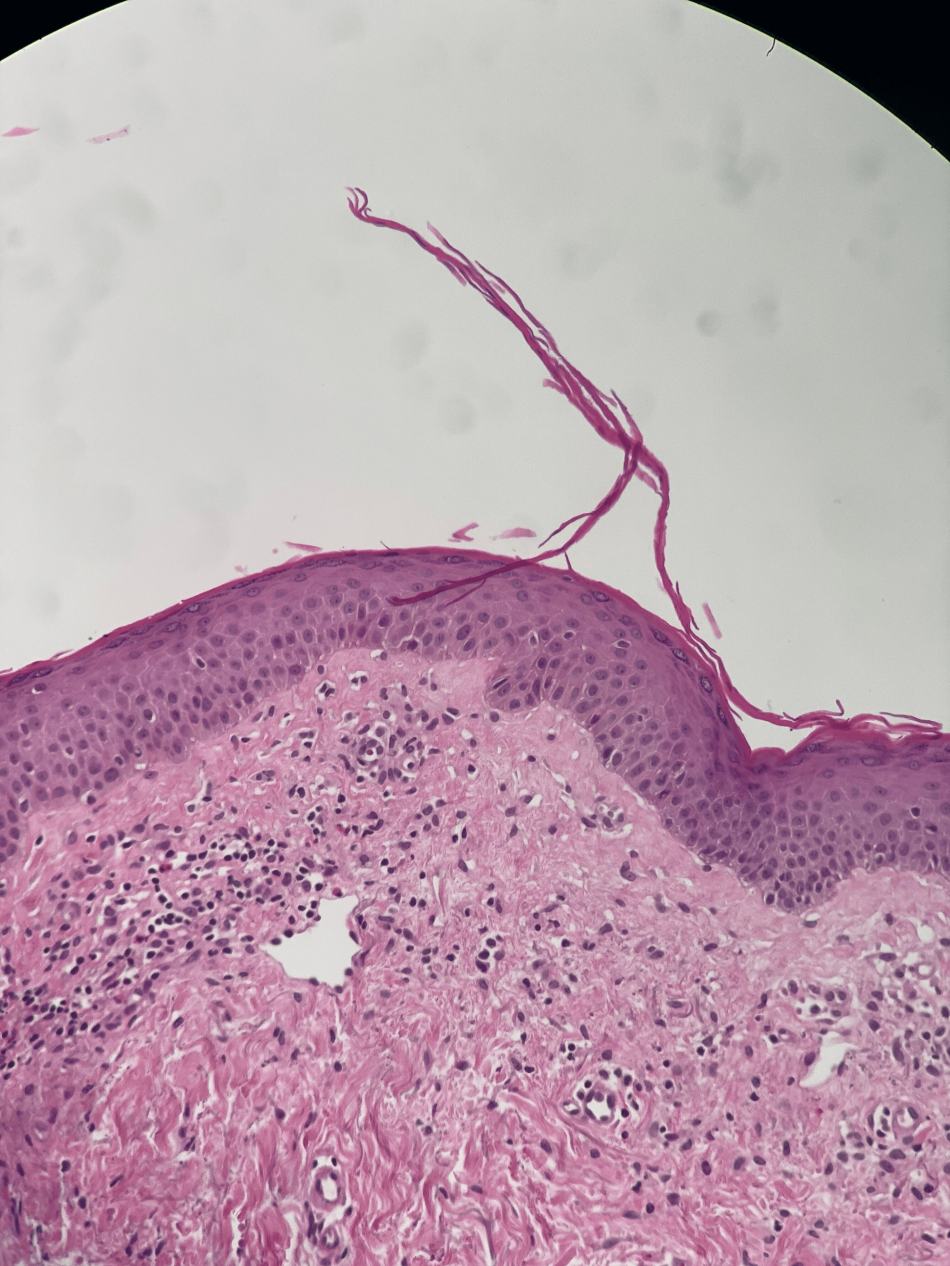
Histopathological features of PRP. Histopathological image showing dilated papillary dermal vessels with perivascular lymphocytic infiltrate and notable eosinophils (high magnification). PRP, pityriasis rubra pilaris

The patient presented for a follow-up appointment post-surgery. The surgical pathology report dated February 12, 2023, revealed findings from a 3 mm skin punch biopsy taken from the patient's abdomen, collected due to pruritic papules present all over the body for the past two years. Microscopic examination displayed features consistent with a PRP-like eruption, characterized by broad epidermal ridges with thick suprapapillary plates with few scattered lymphocytes and a mild perivascular lymphocytic infiltrate with eosinophils in the dermis, alongside slightly dilated superficial vessels (Figures 2, 3). The report mentioned a PRP-like eruption and raised a query regarding a potential association with the initiation of insulin therapy or another drug. However, no recent changes in medication were identified. The patient remained on his existing regimen throughout the disease course, including during recovery. Although the initial pathology report described the findings as a *PRP-like eruption*, this term is sometimes used in histopathology to indicate atypical features that are suggestive but not fully diagnostic without definitive clinical correlation. However, as will be detailed later in this report, the clinical presentation, histopathological findings, and sustained response to targeted therapy supported a diagnosis of classic PRP rather than a drug-induced eruption.

The clinical and histological findings shed light on the patient's dermatological condition, guiding subsequent treatment decisions and follow-up evaluations. Subsequently, the patient commenced oral acitretin (25 mg) OD for a month and betamethasone cream (0.1%) applied topically twice daily for one month to manage inflammatory skin lesions.

Seven weeks after initiating the acitretin regimen, the patient reported an improvement in his condition. Laboratory tests conducted revealed normal lipid and hepatic profiles. The treatment plan included continuing acitretin 25 mg orally OD and antihistamines 10 mg OD at bedtime for one month.

The patient's follow-up visit after a month (four weeks) revealed a normal hepatic profile, normal total cholesterol, and a triglyceride level of 114 mg/dL. The patient remained clinically stable with no new flare-ups, and the treatment regimen was continued without modifications. During this visit, urea cream (10%) was added to the treatment regimen to be applied topically OD, along with soft white paraffin twice daily for three months, to mitigate xerosis. Oral acitretin (25 mg) OD was continued for one month. The patient was scheduled for follow-up in one month; however, he missed the appointment and returned approximately 10 weeks later after his last visit.

At this delayed follow-up, about five months since the initial visit, the patient presented with a flare-up of symptoms, which was attributed to a five-week lapse in acitretin use following the end of his last prescribed course. Laboratory tests, including liver function tests (LFT) and total yellow globules (TYG), were within normal limits. The patient continued antihistamines for pruritus management and was restarted on oral acitretin (25 mg) OD to address keratinization abnormalities associated with PRP.

The patient was seen three weeks later after resuming acitretin treatment. Unfortunately, no clinical improvement was observed, and the patient's symptoms had worsened. Although the flare was initially associated with a brief interruption in acitretin therapy, the lack of response despite resumption further supported the need for treatment escalation. Given the limited response over several months, ustekinumab was initiated during this visit as a second-line agent, based on its efficacy in refractory PRP and its favorable safety profile in older adults.

**Figure 4:**
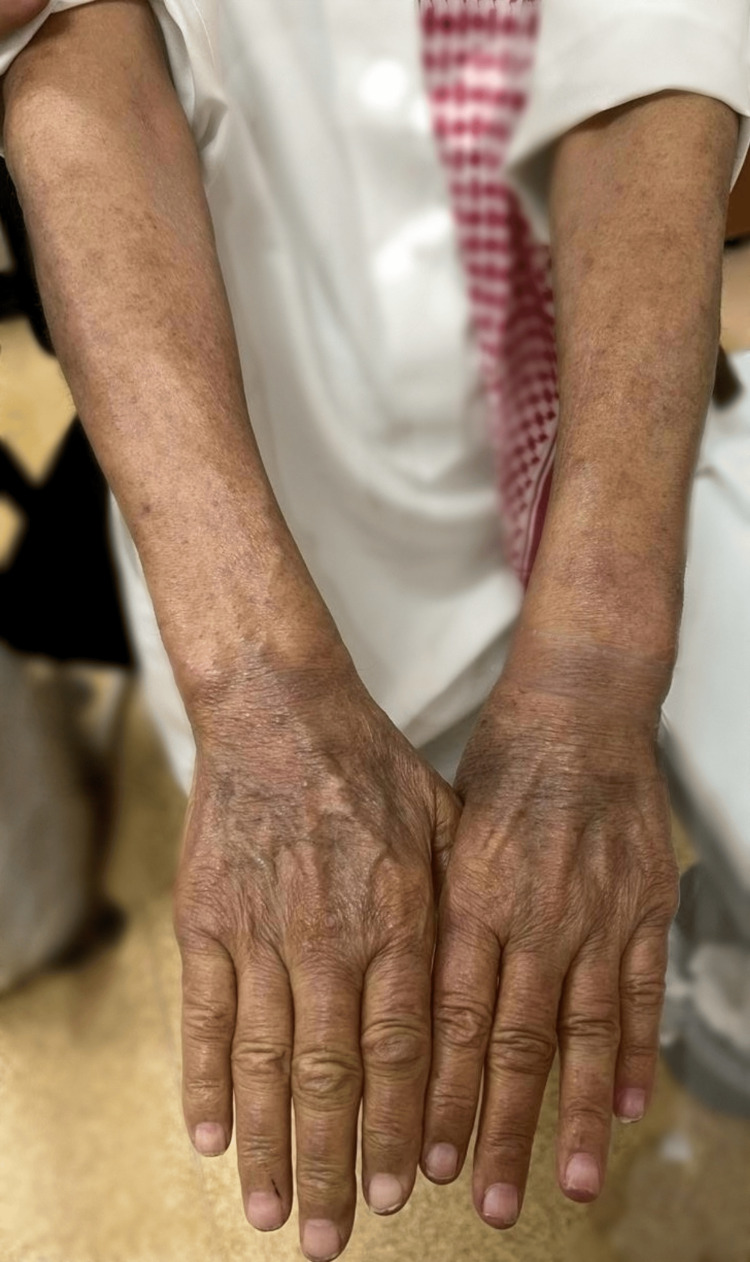
Marked improvement one month after initiating ustekinumab, with resolution of active lesions and residual post-inflammatory hyperpigmentation.

Consequently, ustekinumab therapy 90 mg subcutaneous (SQ) once monthly was administered alongside clobetasol propionate cream (0.05%) applied topically OD for one month to address inflammatory skin lesions, in addition to continued antihistamines for pruritus control. He attended a follow-up appointment one month later since the start of ustekinumab therapy with topical clobetasol propionate. The patient demonstrated clinical improvement (Figure 4). The treatment plan included continuation of ustekinumab therapy along with an oral antihistamine (10 mg once daily) for one month.

Two months after initiating ustekinumab, the patient reported doing well and demonstrated noticeable clinical improvement. However, on examination, severe xerosis and post-inflammatory hyperpigmentation (PIH) were noted. Following this, the patient was prescribed chlorpheniramine maleate syrup (2 mg/5 mL) at a dosage of 4 mL OD at bedtime for symptomatic relief of pruritus, along with topical soft white paraffin applied twice daily to alleviate skin dryness. Follow-up measurements were as follows: blood pressure, 99/56 mmHg; heart rate, 67 bpm; temperature, 35 °C; height, 156 cm; and weight, 65 kg.

The patient attended a follow-up appointment about four and a half months (20 weeks) after the start of ustekinumab. He reported doing well, with notable improvement in xerosis and visible regression of inflammatory lesions, and expressed satisfaction with the progress of his treatment.

During the last visit on March 17, 2024, marking seven months (32 weeks) since the initiation of ustekinumab treatment, the patient's condition showed continued improvement with complete resolution of the lesions. Throughout the biologic therapy course, the patient tolerated ustekinumab well, with no reported adverse effects. Finally, ustekinumab was administered subcutaneously at a dose of 90 mg once monthly, consistent with the treatment regimen since the initiation of therapy.

This case demonstrated a clear timeline of treatment response over one year, beginning with initial presentation in February 2023 and progressing from systemic retinoids to biologic therapy, ultimately resulting in complete resolution by March 2024.

## Discussion

PRP is a rare inflammatory dermatosis characterized by erythematous plaques with follicular hyperkeratotic papules, often involving the entire body surface area. The pathogenesis of PRP remains elusive, with several hypotheses proposed, including genetic predisposition, autoimmune mechanisms, and environmental triggers. In this case, the patient presented with classic clinical features consistent with PRP, including pruritic, purple papules, plaques, and scaly lesions distributed symmetrically on the hands, chest, and legs.

The differential diagnosis of PRP encompasses various dermatological conditions, including mycosis fungoides (MF), psoriasis, lichen planus, and paraneoplastic syndromes. The diagnosis of PRP relies on clinical and histopathological findings. In our case, histological examination revealed features characteristic of PRP, including broad epidermal ridges with thick suprapapillary plates and a mild perivascular lymphocytic infiltrate with eosinophils in the dermis, alongside slightly dilated superficial vessels. This histological pattern is consistent with previous reports of PRP cases [5,6]. Table 2 summarizes the Griffiths and Miralles classifications of PRP, which help distinguish the clinical subtypes [1-3].

**Table 2:** Characteristics of PRP subtypes. Adapted from [1-3]. PRP, pityriasis rubra pilaris

PRP subtype	Notable characteristics
I: Classical adult	Possible erythroderma and palmoplantar keratoderma are indicated by the caudal distribution of reddish-orange keratotic follicular papules that form islands of sparing in plaque formation. Longitudinal ridging, subungual hyperkeratosis, and rough, yellow-brown nails
II: Atypical adult	Susceptibility in the lower extremities, ichthyosiform dermatitis, and potential alopecia
III: Classic juvenile	Comparable to type I, but usually starting between the ages of 5 and 10
IV: Circumscribed juvenile	Clearly defined regions of erythema and follicular hyperkeratosis across the knees and elbows, indicating a less favorable prognosis compared to type III
V: Atypical juvenile	Early onset with a prolonged course. Possible sclerodermatous alterations on the hands and feet secondary to ichthyosiform dermatitis
VI: HIV associated	An HIV-positive individual with acne conglobata, lichen spinulosus-like lesions, hidradenitis suppurativa, and a history of more frequent erythroderma

Treatment of PRP poses significant challenges due to its chronic and relapsing nature. The therapeutic approach varies depending on disease severity, patient characteristics, and response to previous treatments. Systemic retinoids, such as acitretin, have effectively managed PRP by modulating keratinocyte proliferation and differentiation [4]. Our patient showed partial improvement with acitretin therapy; however, due to persistent symptoms, ustekinumab, a monoclonal antibody targeting interleukin-12 and -23, was initiated, resulting in significant clinical improvement [7].

In cases of refractory PRP, biologic agents targeting specific cytokines implicated in disease pathogenesis have shown promising results. Risankizumab, a selective inhibitor of interleukin-23, has been successfully used to treat PRP in children, highlighting its potential in managing severe cases [8]. Similarly, ixekizumab, a monoclonal antibody targeting interleukin-17A, rapidly cleared PRP lesions in an HIV-positive patient, underscoring the role of targeted immunotherapy in challenging cases [9].

Ustekinumab was selected over other biologics due to its established efficacy in refractory PRP and a favorable safety profile in older adults [7]. Although IL-17 and IL-23 inhibitors such as ixekizumab and risankizumab have shown promise, particularly in resistant or pediatric cases [8,9], ustekinumab’s broader immunologic target (IL-12/23) and its proven long-term tolerability made it a suitable second-line agent in this case. Despite initial partial response, the recurrence of symptoms and lack of improvement following the reintroduction of acitretin suggested emerging therapeutic resistance [4]. This clinical course supported the need for timely escalation to biologic therapy.

This case highlights several important clinical considerations in the management of PRP. First, the importance of histopathological examination in confirming the diagnosis and guiding treatment decisions cannot be overstated, particularly in cases with atypical clinical presentations or overlapping features with other dermatoses. Second, the sequential escalation of therapy from topical agents to systemic agents, including retinoids and biologics, underscores the need for a tailored approach based on disease severity and response to treatment. Finally, the successful outcome observed with ustekinumab therapy suggests its potential utility as a promising treatment option for refractory cases of PRP, although further studies are warranted to elucidate its long-term efficacy and safety profile in this context.

## Conclusions

In conclusion, this case highlights the complexity of managing PRP, with histopathological confirmation playing a key role in diagnosis and therapeutic planning. It demonstrates the effectiveness of ustekinumab in treating refractory PRP after an incomplete response to acitretin. The patient’s gradual, sustained improvement supports the consideration of interleukin-targeting biologics as a viable option for managing severe or treatment-resistant PRP. With a clearly documented one-year treatment course, this case reinforces the value of stepwise escalation in PRP management. Continued research is needed to identify targeted therapeutic strategies to improve treatment outcomes and enhance the quality of life for affected individuals.
